# Supplementary material for: Ultralarge anti-Stokes lasing through tandem upconversion
Source: Nat Commun. 2022 Feb 24;13:1032. doi: 10.1038/s41467-022-28701-1 (PMC8873242; doi:10.1038/s41467-022-28701-1)
Supplement: Supplementary file 1 — Supplementary Information [file 41467_2022_28701_MOESM1_ESM.docx]

Supplementary Information

Ultralarge Anti-Stokes Lasing through Tandem Upconversion

*Tianying Sun^1,2,3†^, Bing Chen^1,3†^, Yang Guo^1,3^, Qi Zhu^1,3^, Jianxiong Zhao^1,3^, Yuhua Li^4^, Xian Chen^5^, Yunkai Wu^6^, Yaobin Gao^2^, Limin Jin^6^*, Sai Tak Chu^4^* and Feng Wang^1,3^**

^1^Department of Materials Science and Engineering, City University of Hong Kong, 83 Tat Chee Avenue, Hong Kong SAR, China

^2^School of Chemical Engineering and Technology, Sun Yat-sen University, Zhuhai 519082, China

^3^City University of Hong Kong Shenzhen Research Institute, Shenzhen 518057, China

^4^Department of Physics, City University of Hong Kong, 83 Tat Chee Avenue, Hong Kong SAR, China

^5^College of Materials Science and Engineering, Shenzhen University, Shenzhen 518060, China

^6^State Key Laboratory on Tunable laser Technology, Ministry of Industry and Information Technology Key Lab of Micro-Nano Optoelectronic Information System, Harbin Institute of Technology (Shenzhen), Shenzhen 518055, China

^†^These authors contributed equally to this work

*Email: [jinlimin@hit.edu.cn](mailto:jinlimin@hit.edu.cn) (L.J.); [saitchu@cityu.edu.hk](mailto:saitchu@cityu.edu.hk) (S.T.C.); [fwang24@cityu.edu.hk](mailto:fwang24@cityu.edu.hk) (F.W.)

**I. Supplementary Methods**

**1. Reagents.** Y(CH_3_CO_2_)_3_·xH_2_O (99.9%), Yb(CH_3_CO_2_)_3_·xH_2_O (99.9%), Tm(CH_3_CO_2_)_3_·xH_2_O (99.9%), Er(CH_3_CO_2_)_3_·xH_2_O (99.9%), Ce(CH_3_CO_2_)_3_·xH_2_O (99.9%), Lu(CH_3_CO_2_)_3_·xH_2_O (99.9%), NaOH (>98%), NH_4_F (>98%), trifluoroacetic acid (CF_3_COOH), sodium trifluoroacetate (CF_3_COONa), 1-octadecene (ODE, 90%), oleic acid (OA, 90%) were all purchased from Sigma-Aldrich. Absolute ethanol (99.85%), methyl alcohol (99.99%), and cyclohexane (99.9%) were purchased from VWR International. All chemicals were used as received without further purification.

**2. Synthesis of NaYF_4_:Yb/Tm core nanoparticles.** The NaYF_4_:Yb/Tm (40/1%) core nanoparticle was synthesized by using our previously established protocol^1^. Firstly, 4 mL of RE(CH_3_CO_2_)_3_ (0.2 M, RE = Y, Yb, and Tm) aqueous solution was added to a binary solvent mixture of OA (6 mL) and ODE (14 mL) in a 50 mL flask. The mixture was heated at 150 °C for 50 min before cooling down to 45 °C. Shortly thereafter, 7.9 mL of NH_4_F (0.4 M) in methanol solution and 2 mL of NaOH (1 M) in methanol solution were added, and the resultant solution was stirred for 90 min. After the methanol was evaporated, the solution was heated to 290 °C under argon protection for 1 h and then cooled down to room temperature. The resulting nanoparticles in the mixture were precipitated by the addition of ethanol, collected by centrifugation at 6000 rpm for 3 min, washed with ethanol and methanol for several times, and finally re-dispersed in 4 mL cyclohexane for further use.

**3. Preparation of shell precursor solution.** The preparation of the NaYF_4_:Ce/Er and NaYF_4_ shell precursor solution started with the preparation of RE(CF_3_COO)_3_ aqueous solution. In a typical synthesis, 45 mL of 0.2 M RE(CH_3_COO)_3_ was added to 10 mL of Na_2_CO_3_ aqueous solution (1.5 M). The resulting precipitate was washed twice with deionized water followed by adding 5 mL of CF_3_COOH to fully dissolve the precipitate. The total volume was then fixed to 45 mL by adding deionized water to form the RE(CF_3_COO)_3_ aqueous solution (0.2 M). The shell precursor solution was formed by adding 4 mL of RE(CF_3_COO)_3_ (RE= Y, Er, Ce or RE = Y; 0.2 M) solution and 2 mL of CF_3_COONa solution (0.4 M) into a binary solvent mixture of OA (10 mL) and ODE (10 mL). The slurry was then heated to 125 °C to remove water for 1 h and vacuumed for another 30 min before cooling down to room temperature.

**4. Synthesis of NaYF_4_:Yb/Tm@NaYF_4_:Ce/Er@NaYF_4_ core−shell−shell nanoparticles.** For the synthesis of core−shell structure, the as-prepared 0.4 mL of core nanoparticle in cyclohexane and 2 mL of interlayer precursor was added to a mixture of OA (1 mL) and ODE (9 mL) under stirring. The mixture was heated to 110 °C for 30 min followed by vacuuming for 10 min to remove the cyclohexane and oxygen. The reaction was then heated to 300 °C for 40 min under argon protection and then cooled down to room temperature. The resulting core−shell nanoparticles in the mixture were precipitated by the addition of ethanol, collected by centrifugation at 6000 rpm for 3 min, washed with ethanol and methanol for several times, and finally re-dispersed in 2 mL of cyclohexane for the later outmost shell coating. The coating of the outmost NaYF_4_ shell can follow the same procedure, except for using the NaYF_4_ shell precursor and use all the as-prepared core−shell nanoparticles as the seeds. The same procedure of coating NaYF_4_ shell was repeated once again to ensure thicker NaYF_4_ shell protection.

**5. Synthesis of ligand-free nanoparticles.** The as-prepared core−shell nanoparticles in cyclohexane (2 mL) were extracted and re-dispersed in 3.2 mL of HCl solutions (0.05 M). The slurry solution was then sonicated at room temperature for 1 h and kept still overnight to remove the surface oleate ligands. After the reaction, the ligand layer was discarded, and the nanoparticles were collected *via* centrifugation at 14000 rpm for 30 min and re-dispersed in ethanol. The washing process was repeated twice, and the ligand-free nanoparticles were finally re-dispersed in 2 mL of ethanol.

**6. Materials characterization.** Powder X-ray diffraction (XRD) analysis was performed on a Bruker AXS D2 phaser with Cu Kα radiation (λ = 1.5406 Å). Transmission electron microscopy (TEM) images were taken on an FEI/Philips Tecnai12 TEM at an acceleration voltage of 120 kV. The upconversion emission spectra were recorded with Ocean Optics USB 2000 and Maya 2000 PRO spectrometers. The decay curves were recorded by an Edinburgh FLS980 spectrometer. Optical micrographs of all the devices were recorded with an advanced research microscope (ECLIPSE Ni-U, Nikon). All measurements were performed at room temperature.

**7. Fabrication of the waveguide structure platform.** The waveguide circuit platform was fabricated from a high refractive index glass. The waveguide core is made of low-loss, high index doped silica glass that is semi-buried within a SiO_2_. The waveguide dimensions and refractive indices are 0.9 μm x 1.5 μm, *n* = 1.70. In the fabrication, high-index silica-glass films were first deposited using standard chemical vapor deposition. Subsequently, waveguides were formed using photolithography and reactive ion etching, producing extremely smooth sidewalls. The waveguides were then buried in fused silica glass. To allow strong interaction between the nanoparticles and the optical fields in the waveguide, the top cladding of the device was removed by chemical-mechanical polishing to the top of the core waveguide.

**8. Excitation of nanoparticles with the waveguide structure.** The nanoparticles in an ethanol dispersion were deposited on the surface of the pigtailed device by drop-casting and allowed the full evaporation of ethanol. The excitation energy pumped by a tunable laser (81960A, Agilent) first goes through a 99:1 coupler which was utilized to assist reading 1% of the input power at 1550 nm before the laser finally arrived at the input port of the waveguide structure. The power at the output was also monitored by a power meter.

**9. Fabrication of suspended silica microdisk.** The suspended silica microdisk array was fabricated through a combination of standard photolithography followed by two-step anisotropic etching (C_4_F_4_) and isotropic etching (SF_6_) process. At the very beginning, the oxidized silicon wafer with a 2-μm-thick oxide layer was diced into small chips (1×1 cm), and then cleaned with acetone, isopropanol, deionized water and oxygen plasma in sequence. Then a 2-μm-thick AZ2020 negative photoresist layer was spin-coated onto the top surface of wafer and patterned with standard photolithography (SVC model H94-25C, 10 μW cm^-2^, 25 sec). Through 75 s development in AZ300MIF, a circular pattern was obtained and transferred onto the oxide layer via inductively coupled plasma (ICP, Oxford Plasmalab System100 ICP 180) anisotropic etching under C_4_F_4_. Finally, an isotropic etching procedure was performed on the above silica micro for suspended silica microdisk using SF_6_.

**10. Fabrication of microresonator incorporated with DU nanoparticles.** A silica sol-gel film doped with DU nanoparticles was first made by using an acid-catalyzed hydrolysis-condensation reaction approach. Specifically, tetraethyl orthosilicate (TEOS) is added to an ethanol solvent (TEOS:ethanol = 1:1 in volume ratio), followed by water, acetic acid (CH_3_COOH) and DU nanoparticles under vigorous stirring. The molar ratio between TEOS, water, CH_3_COOH and upconversion nanoparticles was held constant (1:10:0.5:0.005). After mixing for 4 h, the solution was aged for 1 h at room temperature. Such gel is deposited on the suspended silica microdisk using a spin coater at 4000 rpm for 40 s. Two distinct annealing steps were performed. First, low-temperature sintering in a tube furnace was conducted to densify the film and remove organic components (80 °C for 60 min, 1 °C min^-1^). Then the film was further calcinated at 400 °C for 60 min (1 °C min^-1^) to obtain amorphous silica film doped with UCNPs. This deposition and annealing process was repeated several times, resulting in films that were approximately 0.3 μm thick. The resultant DU-incorporated microdisk array was reflowed by a CO_2_ pulse laser (CWQ800K) irradiation. The melting boundary of the silica microdisk would shrink into a microresonator with an ultra-smooth surface.

**11. Lasing characterization.** A 1550 nm pulsed laser (pulse width 6 ns, repetition rate 10 Hz, Φ8 mm) was directly focused onto the top surface of the UCNPs-doped microresonator. The emission light from the boundary of the cavity was collected by an optical fiber coupled to an iHR-320 (Horiba) monochromator attached with a photomultiplier tube. The resolution of the spectrometer is 0.06 nm. The *Q*-factor was given to demonstrate the interface quality of the microresonator since whispering gallery modes occur near the interface of the volume with its surroundings. In the coupling system, a single-mode semiconductor tunable laser was used to excite the microresonator through a tapered fiber. Note that the waist of the tapered fiber is ~1 μm. The axial direction of the fiber was kept along with the equatorial plane of the resonator. By scanning the pumping wavelengths, the transmission spectra were recorded by an oscilloscope. Following the equation *Q* = *λ*/Δ*λ* (*λ* and Δ*λ* are the resonant wavelength and the corresponding full width of half maximum, respectively). We observe *Q* values of over 10^5^ for a typical UCNPs-doped microresonator.

**12. Theoretical modeling of upconversion processes.**

The populations of energy levels in Er^3+^ and Ce^3+^ ions are described approximately by the following rate equations:^2,3^

$\frac{{dN}_{0}}{dt}=-\frac{\sigma_{0}P}{h\nu}N_{0}+R_{1}N_{1}+R_{2}N_{2}+R_{3}N_{3}+R_{4}N_{4}+R_{5}N_{5}$; (eq. S1)

$\frac{{dN}_{1}}{dt}=\frac{\sigma_{0}P}{h\nu}N_{0}+C_{0}N_{2}N_{6}-\frac{\sigma_{1}P}{h\nu}N_{1}-R_{1}N_{1}$; (eq. S2)

$\frac{{dN}_{2}}{dt}=-\frac{\sigma_{2}P}{h\nu}N_{2}-C_{0}N_{2}N_{6}+C_{1}N_{3}N_{6}-R_{2}N_{2};$ (eq. S3)

$\frac{{dN}_{3}}{dt}=\frac{\sigma_{1}P}{h\nu}N_{1}-\frac{\sigma_{3}P}{h\nu}N_{3}+C_{2}N_{4}N_{6}-C_{1}N_{3}N_{6}-R_{3}N_{3}$; (eq. S4)

$\frac{{dN}_{4}}{dt}=\frac{\sigma_{2}P}{h\nu}N_{2}+C_{3}N_{5}N_{6}-C_{2}N_{4}N_{6}-R_{4}N_{4}$; (eq. S5)

$\frac{{dN}_{5}}{dt}=\frac{\sigma_{3}P}{h\nu}N_{3}-C_{3}N_{5}N_{6}-R_{5}N_{5}$; (eq. S6)

$\frac{dN_{6}}{dt}=C_{0}N_{2}N_{6}+C_{1}N_{3}N_{6}+C_{2}N_{4}N_{6}+C_{3}N_{5}N_{6}-R_{6}N_{6}$; (eq. S7)

$\frac{dN_{7}}{dt}=-C_{0}N_{2}N_{6}-C_{1}N_{3}N_{6}-C_{2}N_{4}N_{6}-C_{3}N_{5}N_{6}+R_{6}N_{6}$; (eq. S8)

$N_{0}+N_{1}+N_{2}+N_{3}+N_{4}+N_{5}=N_{Er}$;  (eq. S9)

$N_{6}+N_{7}=N_{Ce}$; (eq. S10)

where *N*_i_ (i = 0–7) denotes the population of lanthanide ions at different energy levels, *N*_Er_ and *N*_Ce_ denote the number density of the Er^3+^ and Ce^3+^, *σ*_i_ (i = 0–3) is the absorption cross-section of Er^3+^ at different energy levels, *R*_i_ (i = 1–6) and *C*_i_ (i = 0–3) denote the rate constants of radiative relaxation and the rate constants of cross-relaxation process, *P* represents the excitation power density, *h* is the Plank’s constant, *ν* is the frequency of excitation laser at 1550 nm. The rate equations were solved numerically using Matlab.

**Supplementary Table 1.** Summary of the constant parameters used in the simulations.^4,5^

|  | **i = 0** | **i = 1** | **i = 2** | **i = 3** | **i = 4** | **i = 5** | **i = 6** |
| --- | --- | --- | --- | --- | --- | --- | --- |
| ***σ*_i_** (cm^2^) | 5.0×10^-21^ | 6.0×10^-21^ | 8.0×10^-21^ | 6.0×10^-21^ | N.A. | N.A. | N.A. |
| ***C*_i_** (cm^3^ s^-1^) | 2.5×10^-18^ | 0.5×10^-17^ | 7.0×10^-16^ | 9.0×10^-16^ | N.A. | N.A. | N.A. |
| ***R*_i_** (s^-1^) | N.A. | 1320 | 660 | 500 | 450 | 500 | 1500 |

**II. Supplementary Figures**


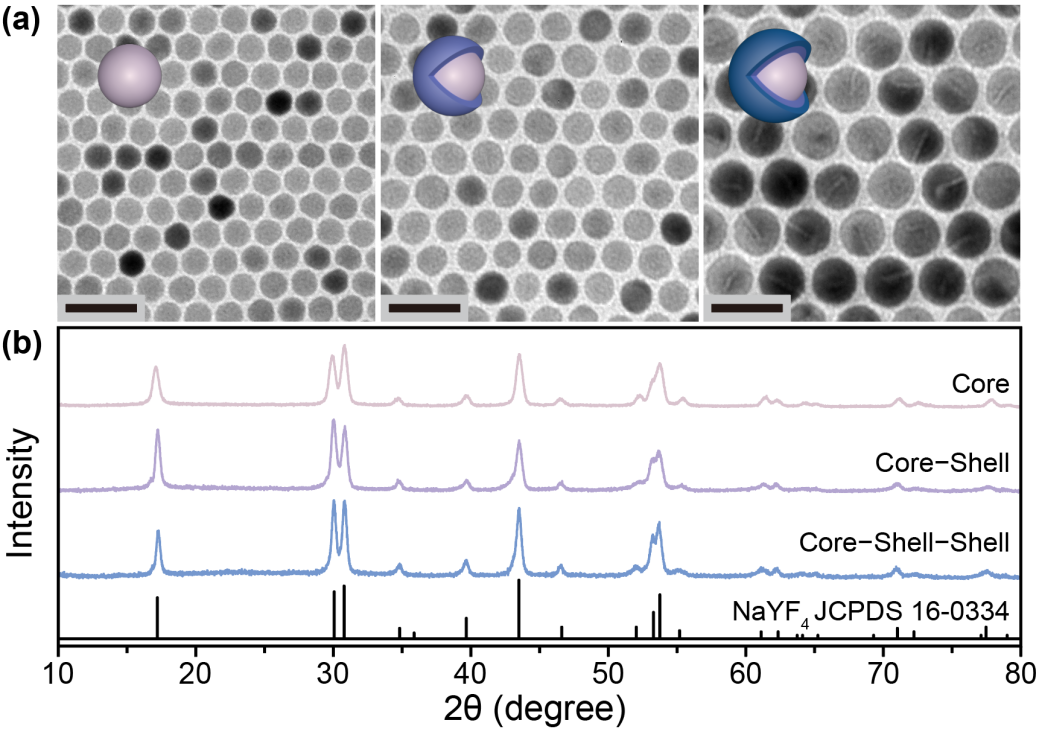


**Supplementary Figure 1. Layer-by-layer growth of NaYF_4_:Yb/Tm@NaYF_4_:Ce/Er@NaYF_4_ core−shell−shell nanoparticles.** (a) TEM images and (b) XRD spectra of the nanoparticles at different stages of the synthesis. Scale bars for TEM images are 50 nm. The line spectrum in (b) is literature data for hexagonal-phase NaYF_4_ (Joint committee on powder diffraction standards file No.16-0334).


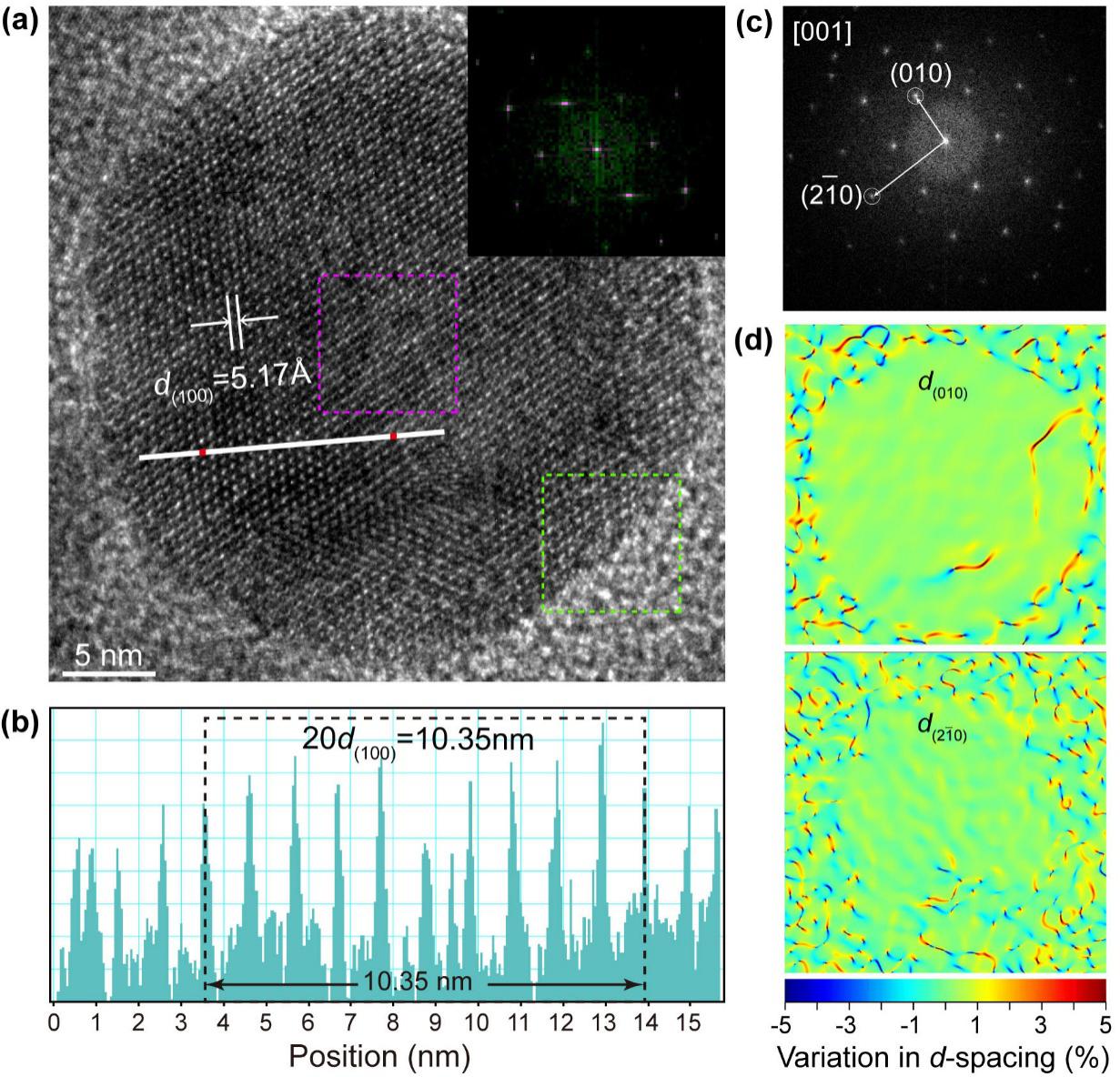


**Supplementary Figure 2. Analysis of crystal lattice in a NaYF_4_:Yb/Tm@NaYF_4_:Ce/Er@ NaYF_4_ core–shell–shell nanoparticle.** (a) HR-TEM image of a typical nanoparticle. Inset: Joint diffractogram obtained by overlapping the Fast Fourier Transform (FFT) patterns for core and shell domains (indicated by the magenta and green squares, respectively) in the nanoparticle. No noticeable splitting of diffraction spots was observed, demonstrating uniform lattice constant across the core/shell interface. (b) Intensity proﬁle recorded by scanning along the line shown in (a), revealing a *d*-spacing of 5.17 Å. (c) FFT pattern of the whole nanoparticle in (a). (d) False-color maps of relative *d*-spacing in the (010) and (2-10) planes, respectively. The *d*-spacing maps were calculated by geometrical phase analysis (GPA) based on the labeled FFT spots in (c).^6^ The results further demonstrate uniform lattice constants in the nanoparticles.

**
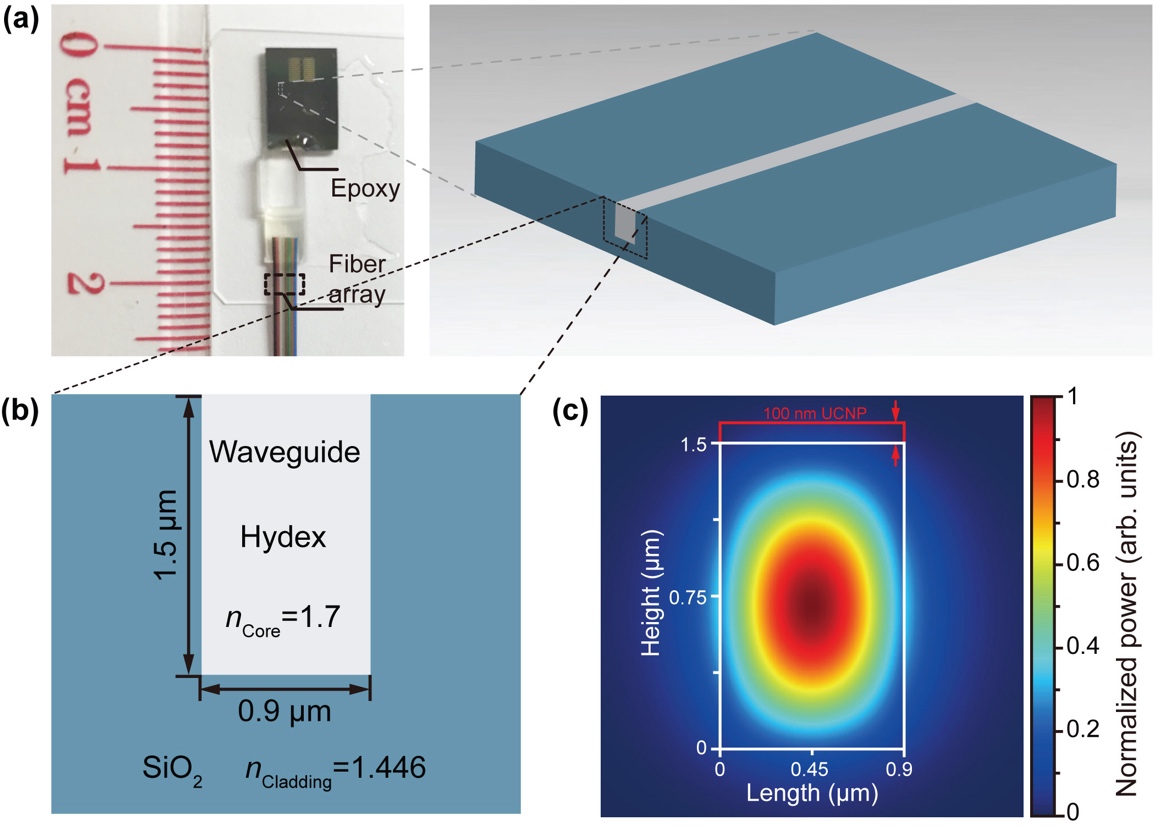
**

**Supplementary Figure 3. The waveguide circuit-based excitation platform.** (a) Photograph and structure of the device used in the experiment. (b) The waveguide circuit configuration. The waveguide (*n* = 1.7) was embedded into SiO_2_ substrate (*n* = 1.446) with the top surface exposed to the environment.^7^ Schematic is not drawn to scale. (c) Simulation of electrical field distribution of the transverse electric (TE) polarized beam in the waveguide structure. According to simulation, 0.6% of total laser power can be received by nanoparticles on top of the waveguide. At an input laser power of 311 mW, the excitation power density received by nanoparticles was estimated to be (311 mW × 0.6%) / (0.9 μm × 100 nm) = 2073 kW cm^-2^.

**
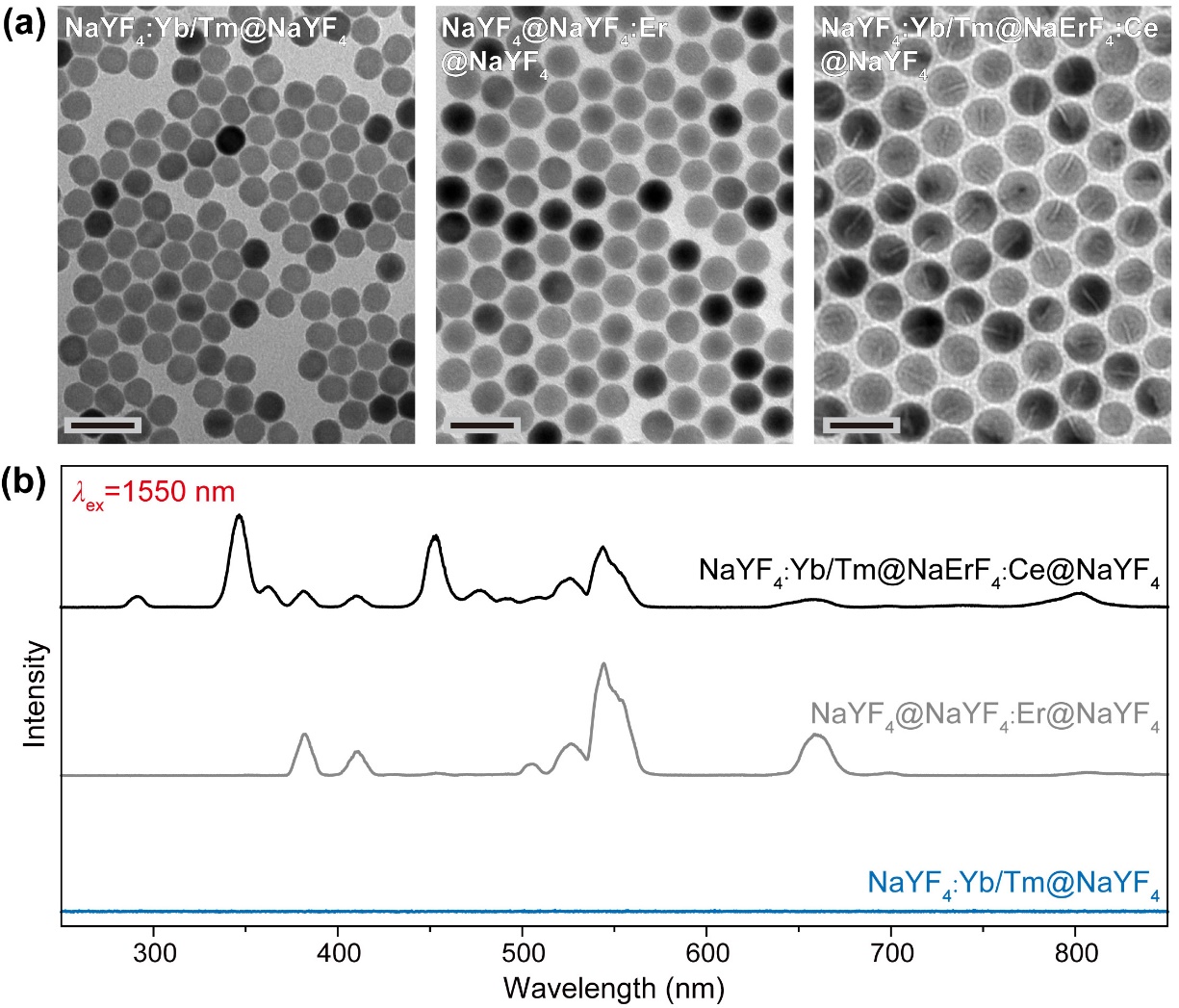
**

**Supplementary Figure 4. Characterization of the NaYF_4_:Yb/Tm@NaYF_4_, NaYF_4_@NaYF_4_:Er@NaYF_4_, and NaYF_4_:Yb/Tm@NaErF_4_:Ce@NaYF_4_ nanoparticles.** (a) TEM images of NaYF_4_:Yb/Tm@NaYF_4_, NaYF_4_@NaYF_4_:Er@NaYF_4_, and NaYF_4_:Yb/Tm@NaErF_4_:Ce@NaYF_4_ nanoparticles, respectively. Scale bars are 50 nm. (b) Emission spectra of NaYF_4_:Yb/Tm@NaYF_4_, NaYF_4_@NaYF_4_:Er@NaYF_4_, and NaYF_4_:Yb/Tm@NaErF_4_:Ce@NaYF_4_ nanoparticles under 1550 nm excitation, respectively. Note that the emission spectrum of NaYF_4_@NaYF_4_:Er@NaYF_4_ nanoparticles was used for reference. The absence of emission peaks from the NaYF_4_:Yb/Tm@NaYF_4_ core–shell nanoparticle indicated that the Yb/Tm-doped upconversion layer does not respond to the 1550 nm excitation.


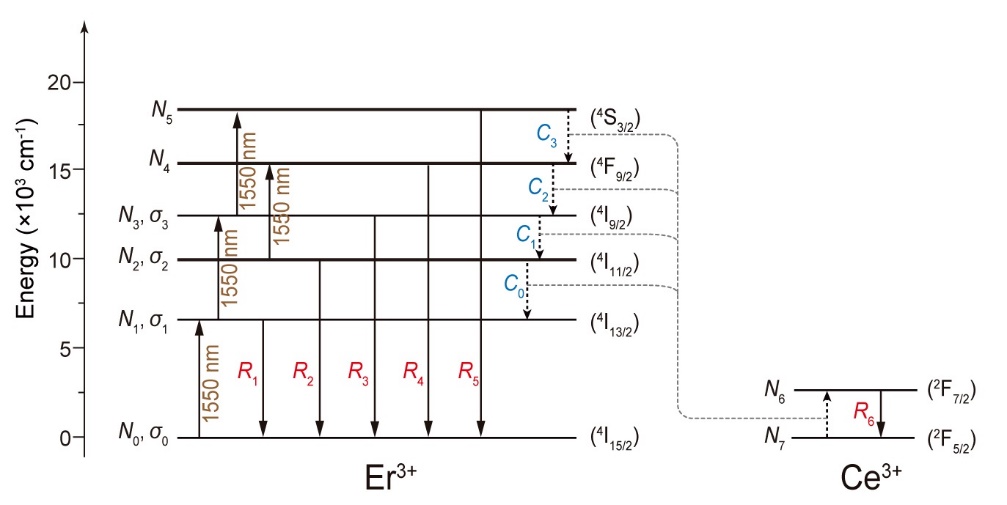


**Supplementary Figure 5. Simplified energy level diagram of Er^3+^ and Ce^3+^ used for the simulation.** The scheme shows ground-state absorption and excited-state absorptions of 1550 nm excitation for the photon upconversion processes, cross-relaxations between Er^3+^ and Ce^3+^ ions, and radiative transitions from different energy levels in the NaYF_4_@NaErF_4_:Ce(30%)@NaYF_4_ nanoparticles.


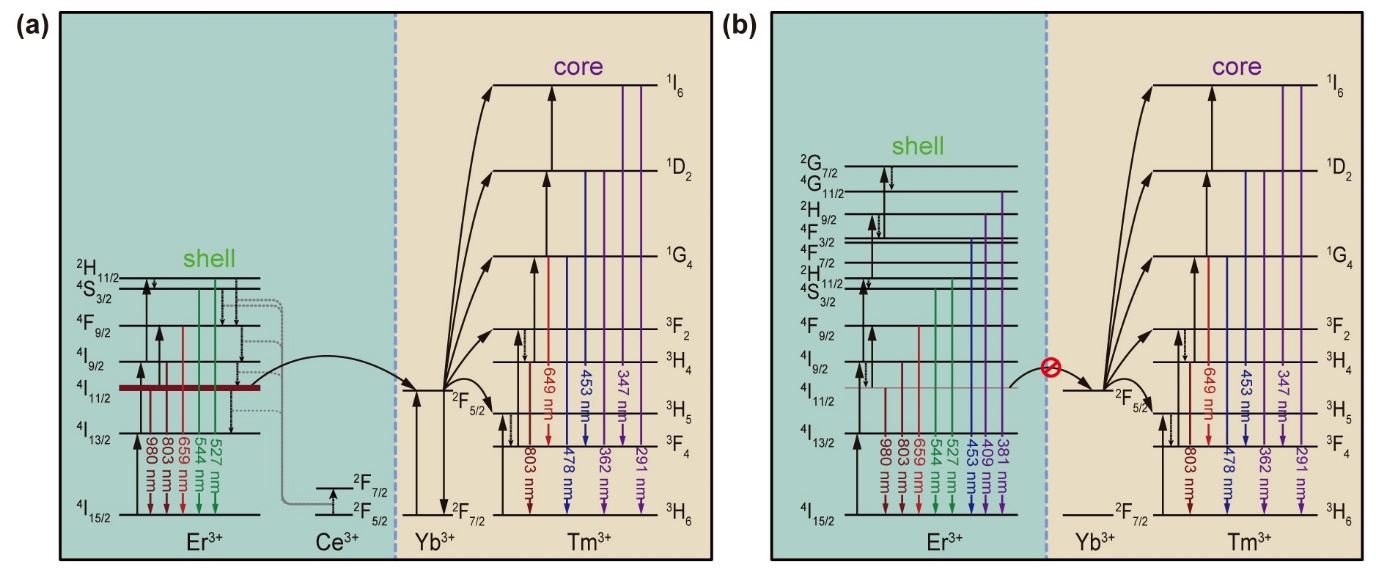


**Supplementary Figure 6. The effect of Ce^3+^ dopant on the energy process.** (a) Proposed energy transfer in NaYF_4_:Yb/Tm@NaErF_4_:Ce@NaYF_4_ nanoparticle. The Ce^3+^ dopant induces selective quenching in Er^3+^ ions through cross-relaxation, thereby facilitating electron population in the ^4^I_11/2_ energy state of Er^3+^ for the subsequent population of ^2^F_5/2_ energy state of Yb^3+^ ions. (b) Proposed energy transfer in NaYF_4_:Yb/Tm@NaErF_4_:Y@NaYF_4_ nanoparticle. Without Ce^3+^ dopant ions, Er^3+^ ions will be straightforwardly excited to the high-lying excited states, which disfavors energy transfer to Yb^3+^ and further to Tm^3+^ ions.

**
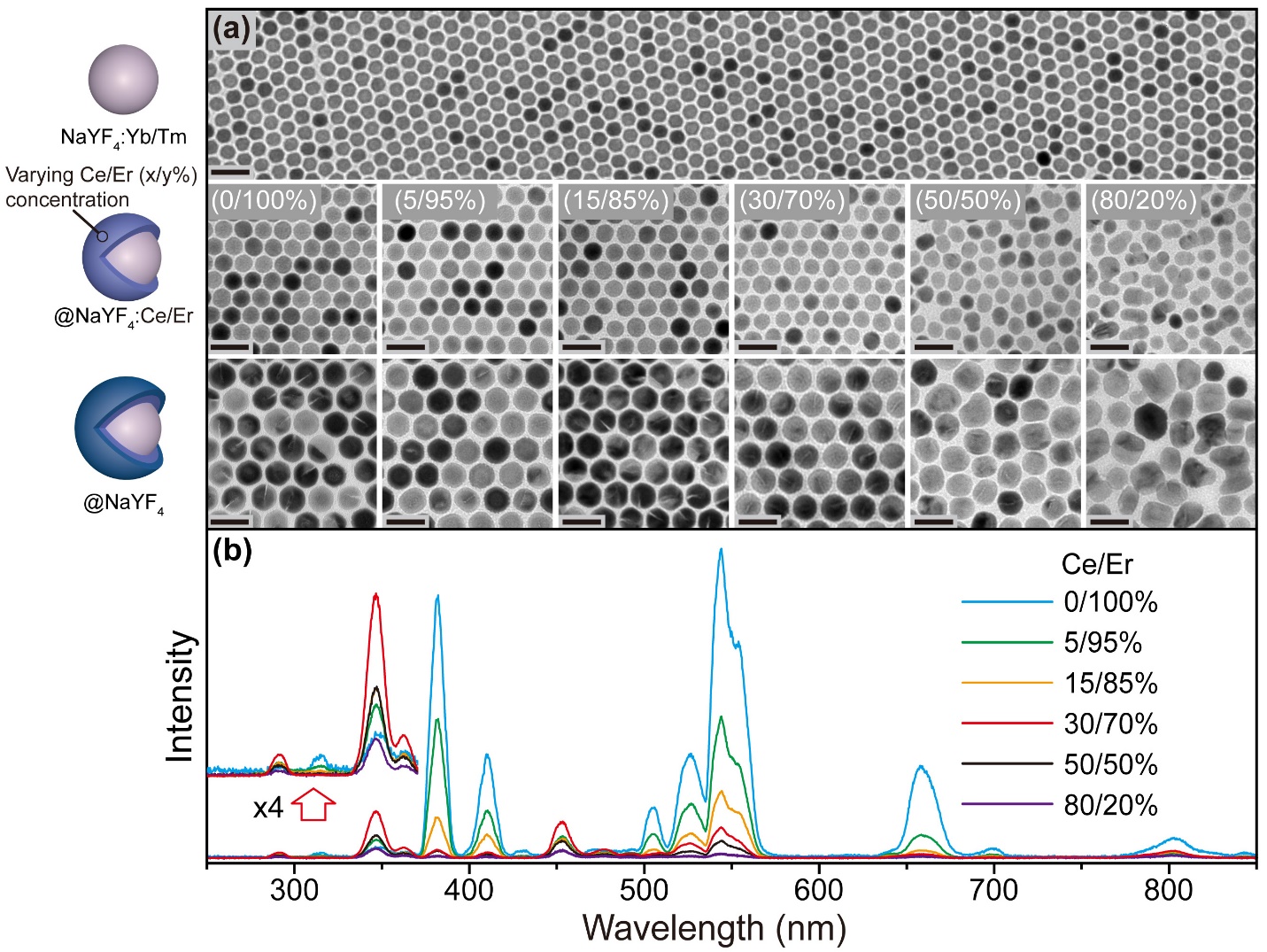
**

**Supplementary Figure 7. Optimization of Ce^3+^/Er^3+^ doping concentration.** (a) TEM images of the nanoparticles doped with varying concentrations of Ce^3+^/Er^3+^. Scale bars are 50 nm. The nanoparticles maintained a uniform shape and size at relatively low Ce^3+^ concentrations (< 30%). Further increase of the Ce^3+^ doping concentration resulted in nonuniform shell growth due to large lattice mismatches between the core, interlayer, and outermost shell layers. (b) Emission spectra of NaYF_4_:Yb/Tm@NaYF_4_:Ce/Er@NaYF_4_ nanoparticles as a function of Ce^3+^/Er^3+^ dopant concentration in the interlayer. Inset: amplified spectra showing the emission in the range of 250-370 nm. The reduction of Tm^3+^ emission intensity at over high Ce^3+^ concentrations (> 30%) was partly ascribed to the nonuniformly coated shell layer.


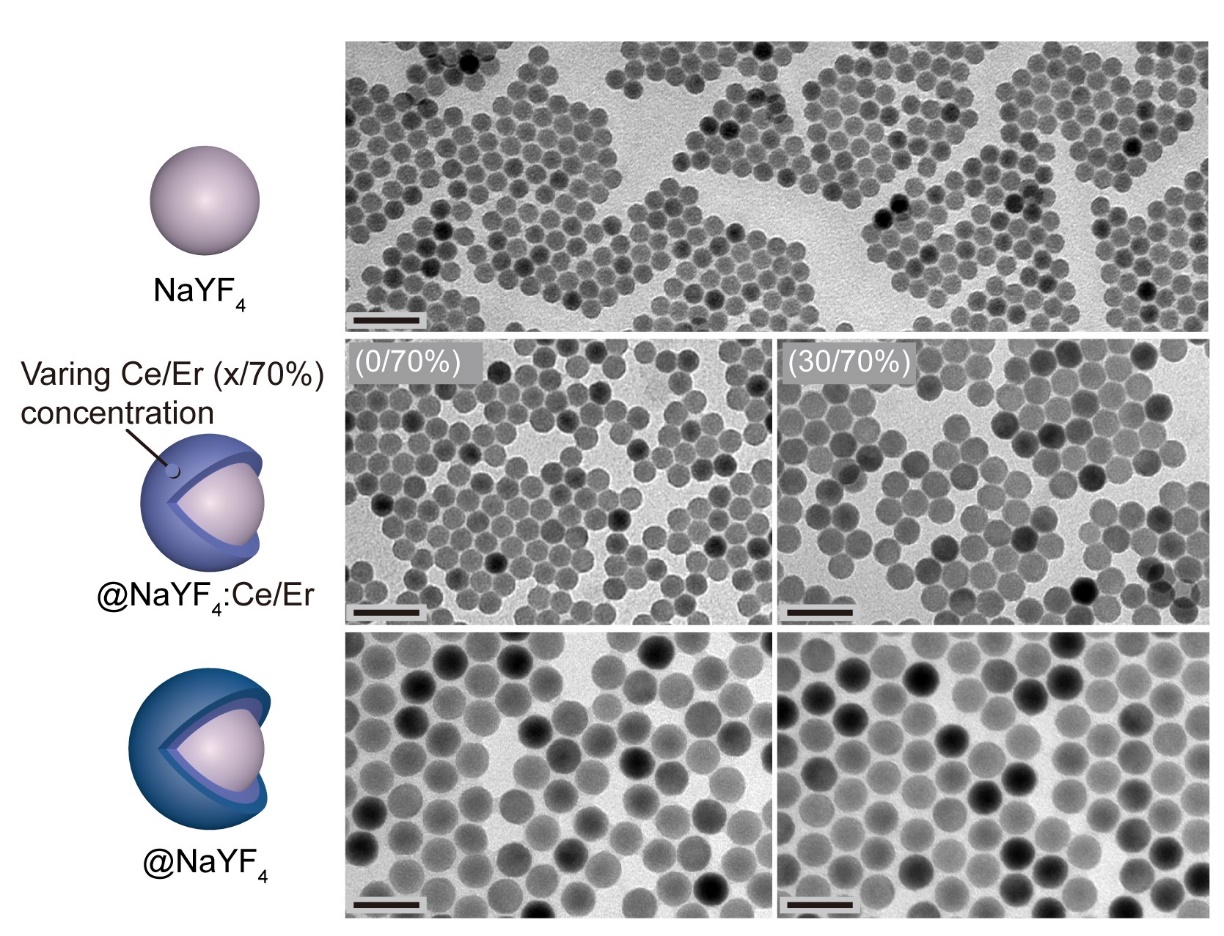


**Supplementary Figure 8. TEM characterization of NaYF_4_@NaYF_4_:Ce/Er@NaYF_4_ core–shell–shell nanoparticles.** Doping of varying concentrations of Ce^3+^ ions (0 & 30%) in the interlayers induced no noticeable changes in the size and morphology of the nanoparticles. Scale bars are 50 nm.


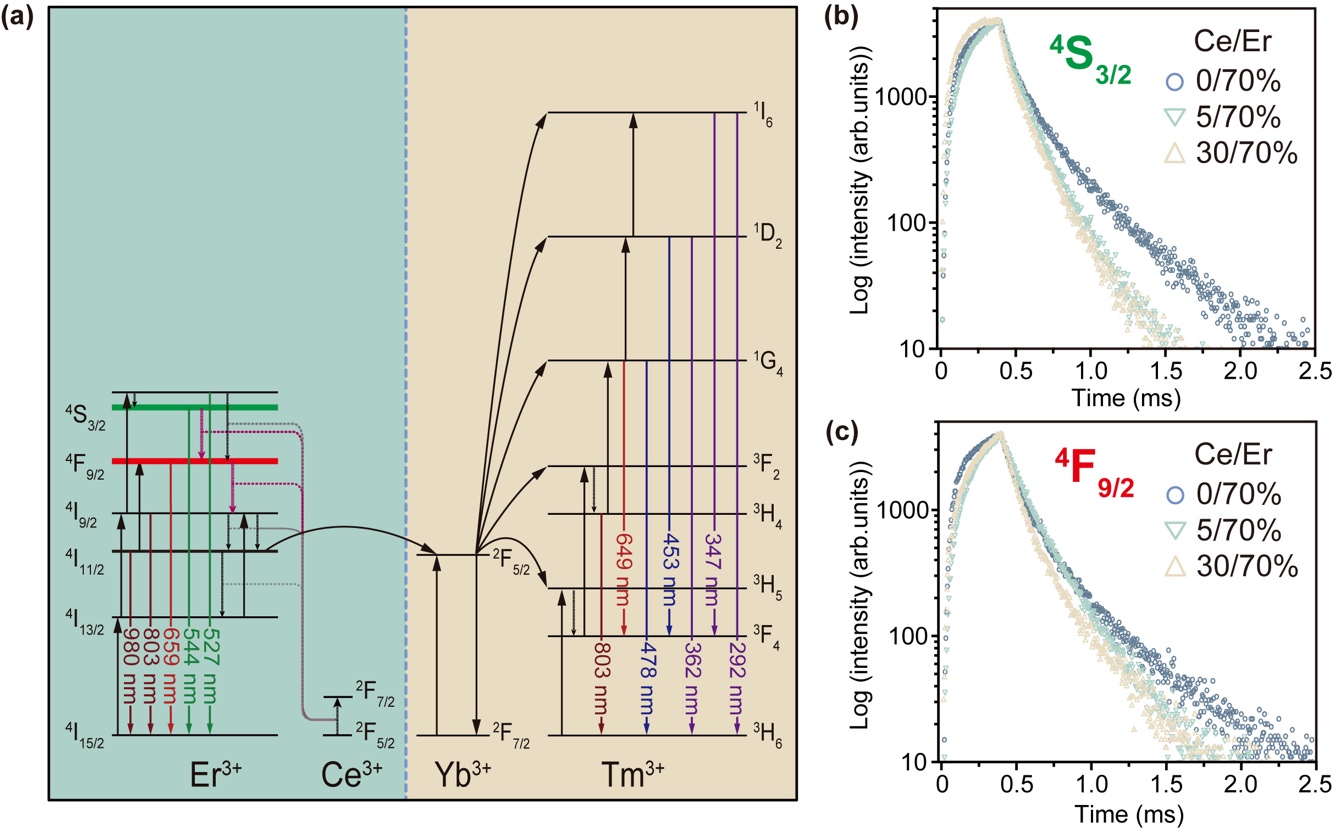


**Supplementary Figure 9. Time-decay study of the NaYF_4_:Yb/Tm@NaYF_4_:Ce/Er@NaYF_4_ core–shell–shell nanoparticles.** (a) Proposed cross-relaxation processes between Er^3+^ and Ce^3+^ for depopulation of the ^4^S_3/2_ and ^4^F_9/2_ states in Er^3+^ ions in NaYF_4_:Yb/Tm@NaErF_4_:Ce@NaYF_4_ nanoparticle. (b, c) Decay curves of ^4^S_3/2_→^4^I_15/2_ and ^4^F_9/2_→^4^I_15/2_ transitions as a function of Ce^3+^ doping concentration.

**
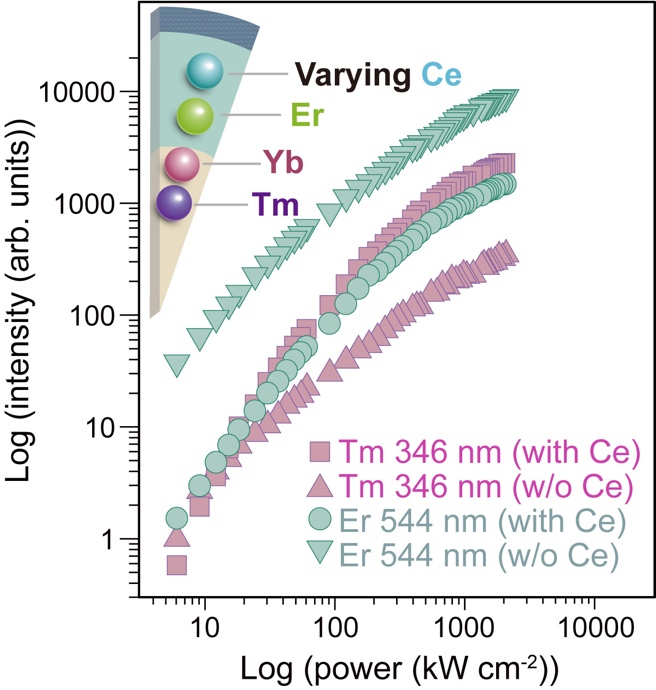
**

**Supplementary Figure 10. Excitation power-dependence of Tm^3+^ and Er^3+^ emission intensities in the NaYF_4_:Yb/Tm@NaYF_4_:(Ce/)Er@NaYF_4_ core–shell–shell nanoparticles.** In the absence of Ce^3+^, the Er^3+^ emission dominated the spectra irrespective of the excitation power, suggesting that the excitation energy was mostly absorbed by the Er^3+^ ions. By contrast, the intensity of the Tm^3+^ emission was greatly promoted with the increase of excitation power and became dominant over that of the Er^3+^ upon the addition of Ce^3+^ ions.


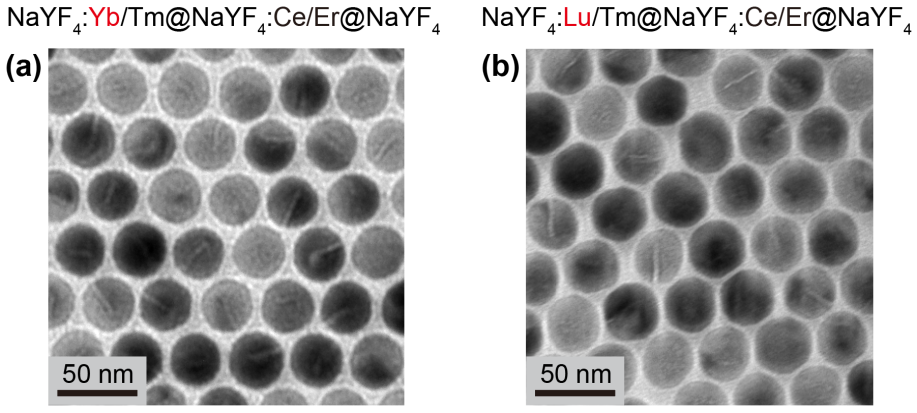


**Supplementary Figure 11. TEM characterization of nanoparticles doped with Yb^3+^ or Lu^3+^ ions in the core.** TEM images of (a) NaYF_4_:Yb/Tm (40/1%)@NaYF_4_:Ce/Er (30/70%)@NaYF_4_ nanoparticles and (b) NaYF_4_:Lu/Tm (40/1%)@NaYF_4_:Ce/Er (30/70%)@NaYF_4_ nanoparticles exhibited similar size and morphology. The observation was as expected because Lu^3+^ ions display a similar ionic radius and comparable chemical property to Yb^3+^ which led to similar reaction processes under the identical synthesis condition.

**
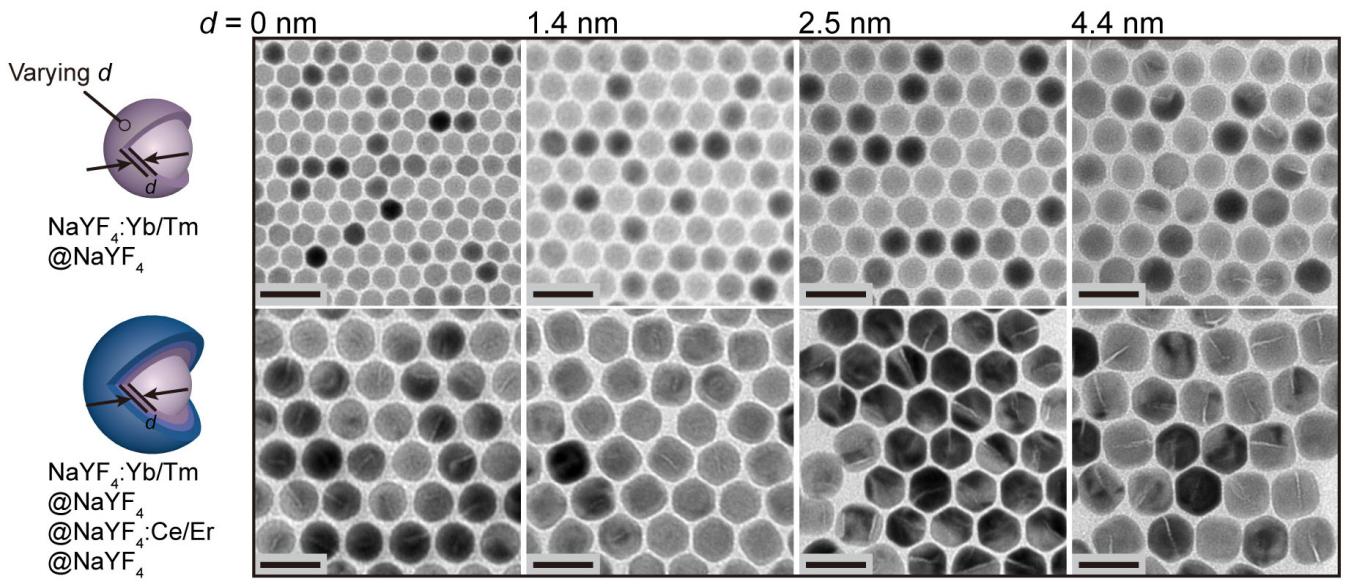
**

**Supplementary Figure 12. Layer-by-layer growth of NaYF_4_:Yb/Tm@NaYF_4_@NaYF_4_:Ce/Er @NaYF_4_ core–shell–shell–shell nanoparticles.** (a) The shell thickness of the NaYF_4_ interlayer was tuned by adding different amounts of NaYF_4_ shell precursor in the synthesis. The mean shell thickness was controlled at 0 nm, 1.4 nm, 2.5 nm and 4.4 nm, respectively. Scale bars are 50 nm.


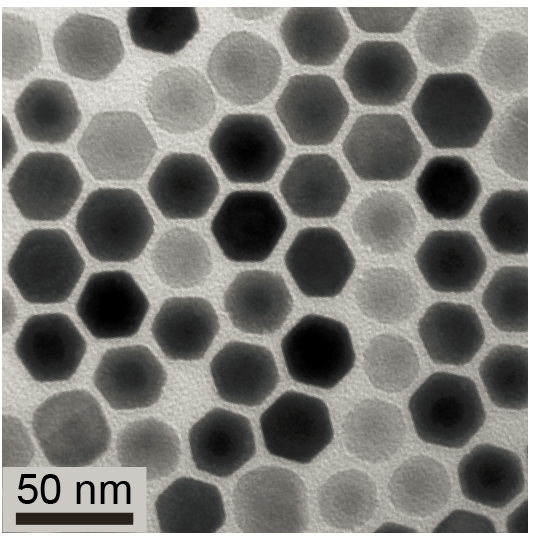


**Supplementary Figure 13. TEM characterization of NaYF_4_:Yb/Tm/Ce/Er (40/1/15/35%)@ NaYF_4_ nanoparticles.** The core**–**shell nanoparticles closely resembled the NaYF_4_:Yb/Tm@ NaYF_4_:Ce/Er@NaYF_4_ core**–**shell**–**shell counterparts.


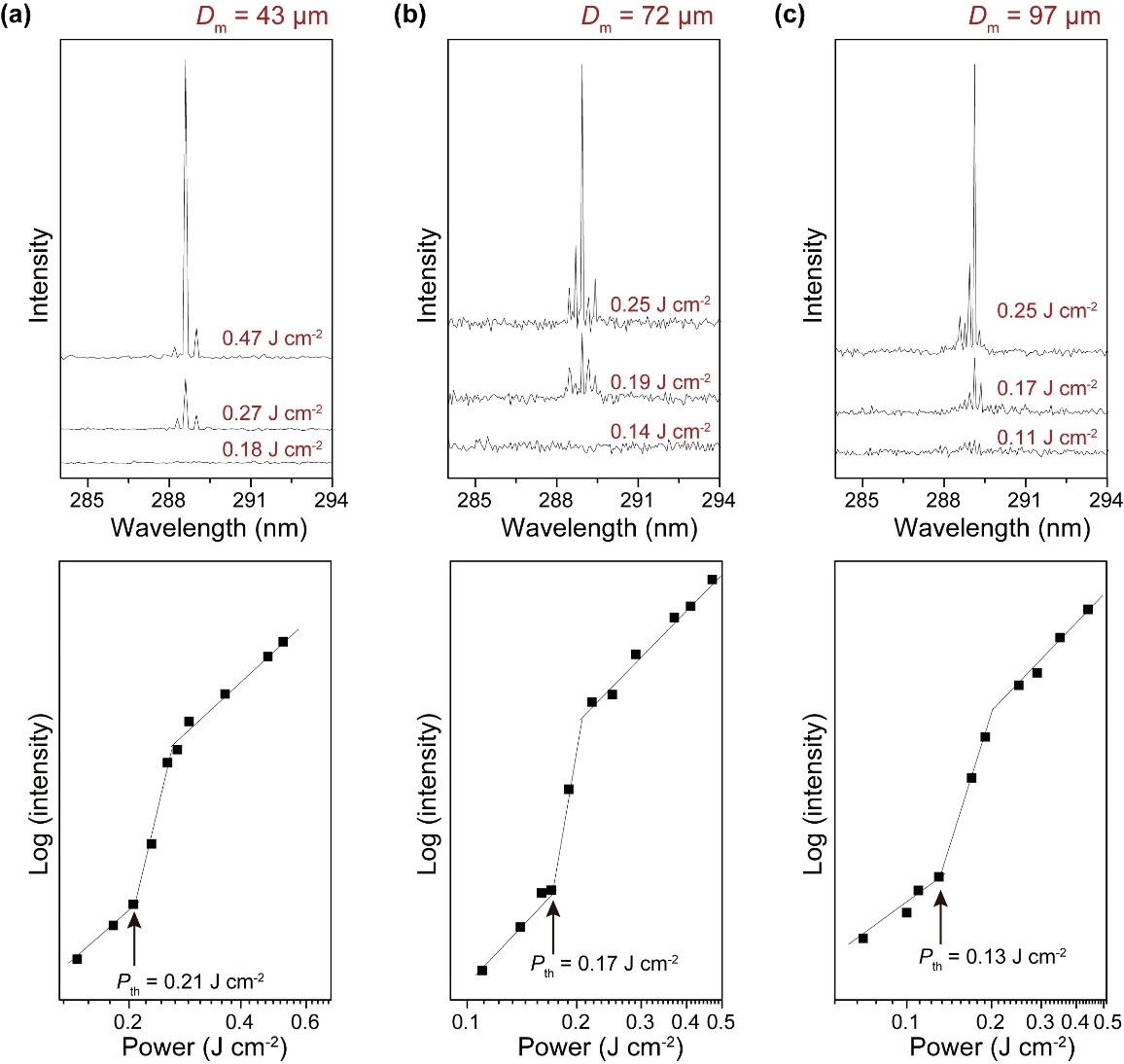


**Supplementary Figure 14. UV lasing in microresonators of different sizes.** Lasing spectra at different excitation powers (top) and logarithmic plot of the output intensity versus excitation power (bottom) in microresonators with (a) *D*_m_ = 43 μm, (b) *D*_m_ = 72 μm and (c) *D*_m_ = 97 μm.


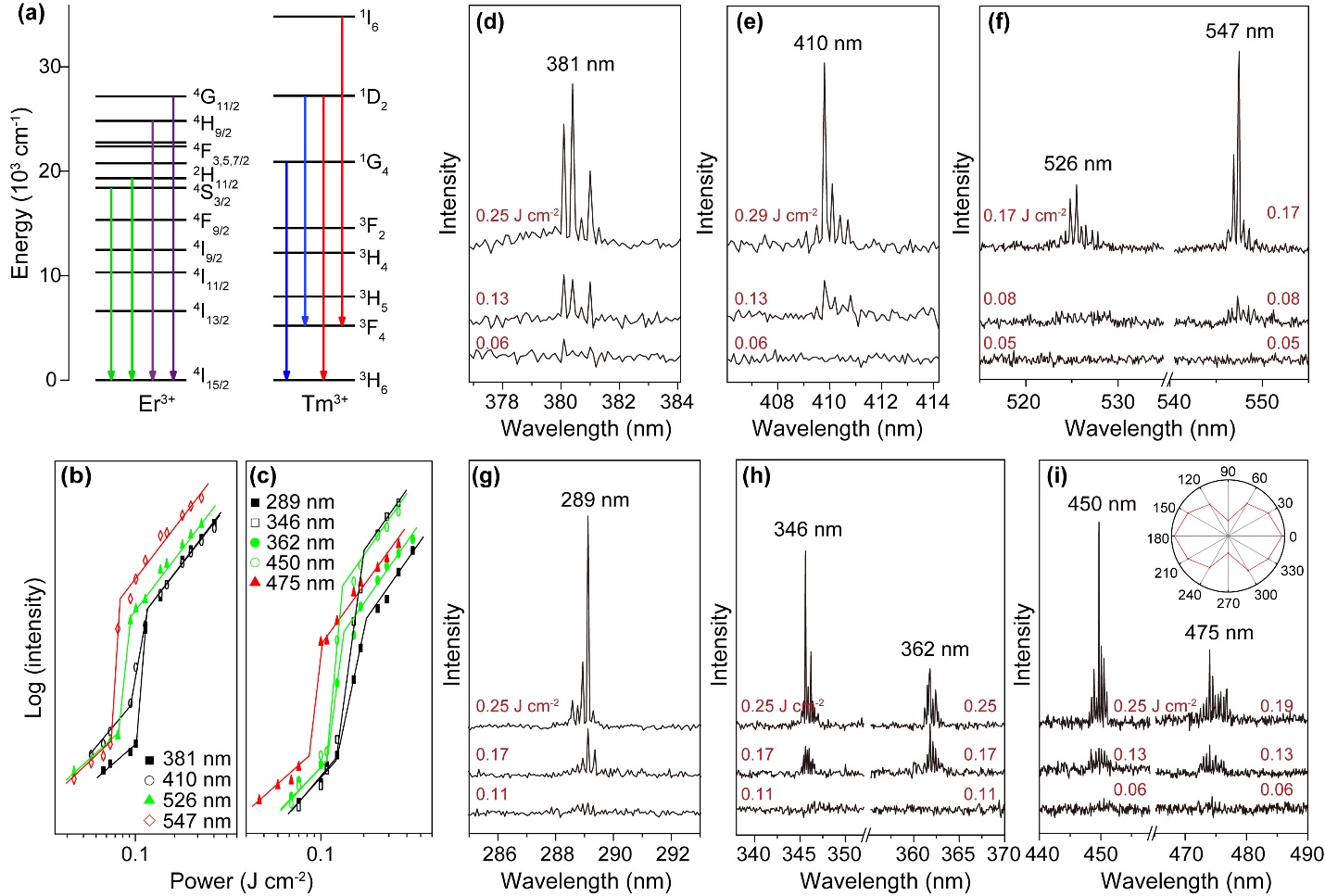


**Supplementary Figure 15. Multi-wavelength lasing from the transitions of Er^3+^ and Tm^3+^ ions in a UCNPs-doped microresonator (~100 μm in diameter).** (a) The simplified energy levels showing the transitions from Er^3+^ and Tm^3+^ ions. (b, c) Logarithmic plot of the output intensities of emissions from Er^3+^ and Tm^3+^ ions versus excitation power for the microresonator, respectively. (d-f) The power-dependent emission spectra at the wavelengths of 381 nm, 410 nm, 526 nm and 547 nm for Er^3+^ ions, respectively. (g-i) The power-dependent emission spectra at the wavelengths of 289 nm, 346 nm, 362 nm, 450 nm, and 475 nm for Tm^3+^ ions, respectively. Note that the lasing thresholds are identified to be *P*_th_ (289 nm) = 0.13 J cm^-2^, *P*_th_ (346 nm) = 0.13 J cm^-2^, *P*_th_ (362 nm) = 0.11 J cm^-2^, *P*_th_ (450 nm) = 0.11 J cm^-2^, *P*_th_ (475 nm) = 0.08 J cm^-2^, *P*_th_ (381 nm) = 0.11 J cm^-2^, *P*_th_ (410 nm) = 0.10 J cm^-2^, *P*_th_ (526 nm) = 0.08 J cm^-2^, and *P*_th_ (547 nm) = 0.07 J cm^-2^, respectively. Inset: the polar plot of the emission at 450 nm. The polarization of lasing was examined by assessing the emission intensity from the side. The UCNPs-doped microring laser was mainly transverse electric (TE, with E in-plane) polarized.


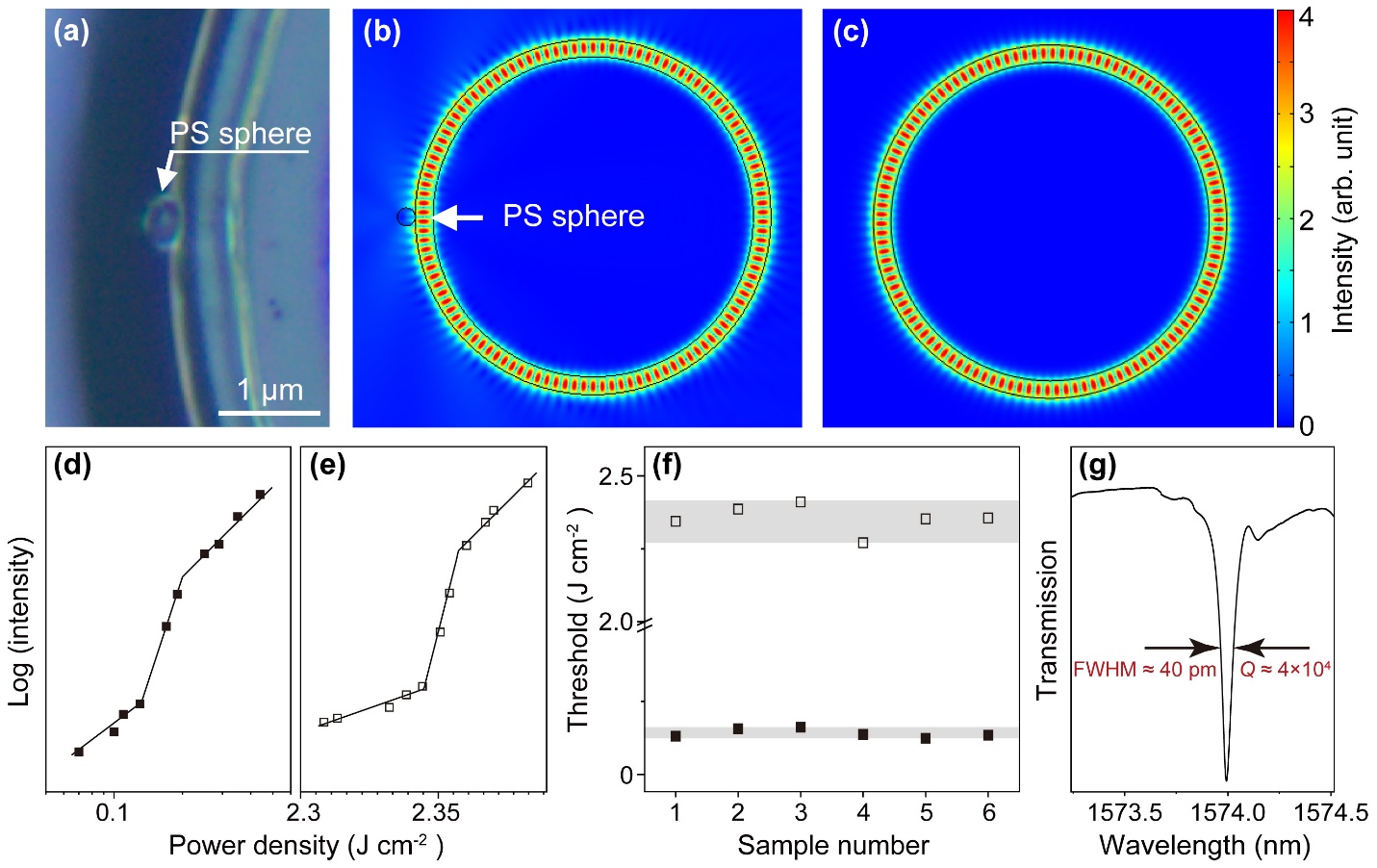


**Supplementary Figure 16. A sensing platform based on the UCNPs-doped toroidal microlaser by monitoring the *P*_th_ change.** (a) Optical image of a PS sphere (300 nm in diameter) attached to the microresonator structure (~100 μm in diameter). The white arrow indicates the PS sphere. (b, c) The simulated field patterns of the excited microresonator with and without PS sphere, respectively. In the calculation, the diameter/width of the microring and the diameter of the PS sphere were set at 4/0.2 μm and 0.2 μm, respectively. The results reveal that deleterious interaction occurs at the joint position between the resonant modes and the target sphere, accompanied by a light escape out of the volume. (d, e) Logarithmic plot of output intensity versus excitation power for the microresonator without and with PS sphere, respectively. (f) Statistics of *P*_th_ values of six microresonators without (bottom panel) and with a PS sphere attached at different points (top panel). (g) Transmission spectra from the UCNPs-doped microresonator attached with an external 300-nm-diameter PS sphere.

**Supplementary References**

1. Wang, F., Deng, R. & Liu, X. Preparation of core-shell NaGdF_4_ nanoparticles doped with luminescent lanthanide ions to be used as upconversion-based probes. *Nat. Protoc*. **9**, 1634 (2014).

2. Wang, Y., Deng, R., Xie, X., Huang, L. & Liu, X. Nonlinear spectral and lifetime management in upconversion nanoparticles by controlling energy distribution. *Nanoscale* **8**, 6666-6673 (2016).

3. Zhan, Q. *et al.* Achieving high-efficiency emission depletion nanoscopy by employing cross relaxation in upconversion nanoparticles. *Nat. Commun.* **8**, 1058 (2017).

4. Daldosso, N. *et al.* Absorption cross section and signal enhancement in Er-doped Si nanocluster rib-loaded waveguides. *Appl. Phys. Lett.* **86**, 261103 (2005).

5. Meng, Z. *et al.* Large improvement in quantum fluorescence yield of Er^3+^-doped fluorozirconate and fluoroindate glasses by Ce^3+^ codoping. *J. Appl. Phys.* **88**, 2187-2190 (2000).

6. Grillo, V. & Rossi, F. STEM_CELL: A software tool for electron microscopy. Part 2 analysis of crystalline materials. *Ultramicroscopy* **125**, 112-129 (2013).

7. Sun, T. et al. Integrating temporal and spatial control of electronic transitions for bright multiphoton upconversion. *Nat. Commun*. **10**, 1811 (2019).
