# Supplementary material for: Ultralarge anti-Stokes lasing through tandem upconversion
Source: Nat Commun. 2022 Feb 24;13:1032. doi: 10.1038/s41467-022-28701-1 (PMC8873242; doi:10.1038/s41467-022-28701-1)
Supplement: Supplementary file 2 — Reporting Summary [file 41467_2022_28701_MOESM2_ESM.pdf]

nature research

Corresponding author(s): *Double-blind peer review submissions: write DBPR and your manuscript number here instead of author names.*

Lasing Reporting Summary

Nature Research wishes to improve the reproducibility of the work that we publish. This form is intended for publication with all accepted papers reporting claims of lasing and provides structure for consistency and transparency in reporting. Some list items might not apply to an individual manuscript, but all fields must be completed for clarity.

For further information on Nature Research policies, including our data availability policy, see Authors & Referees.

~ Experimental design

Please check: are the following details reported in the manuscript?

1. Threshold

Plots of device output power versus pump power over a wide range of values indicating a clear threshold

- [x] Yes
- [ ] No

see Fig. 5(f,h), Supplementary Fig. 14(a-c(bottom ones)) and Fig. 15(b,c).

2. Linewidth narrowing

Plots of spectral power density for the emission at pump powers below, around, and above the lasing threshold, indicating a clear linewidth narrowing at threshold

- [x] Yes
- [ ] No

see Fig. 5(e), Supplementary 14(a-c(top ones)) and Fig. 15(d-f,g-i).

Resolution of the spectrometer used to make spectral measurements

- [x] Yes
- [ ] No

see Lasing Characterization in Section 1 of the Supplementary Information.

3. Coherent emission

Measurements of the coherence and/or polarization of the emission

- [x] Yes
- [ ] No

see the inset in Supplementary Fig. 15(i).

4. Beam spatial profile

Image and/or measurement of the spatial shape and profile of the emission, showing a well-defined beam above threshold

- [ ] Yes
- [x] No

This WGM-based microring lasing emission is inherently isotropic.

5. Operating conditions

Description of the laser and pumping conditions
Continuous-wave, pulsed, temperature of operation

- [x] Yes
- [ ] No

see Lasing Characterization in Section 1 of the Supplementary Information.

Threshold values provided as density values (e.g. W cm^{-2} or J cm^{-2}) taking into account the area of the device

- [x] Yes
- [ ] No

see Fig. 5f, Supplementary Fig. 14(a-c(bottom ones)), and figure description in Supplementary Fig. 15.

6. Alternative explanations

Reasoning as to why alternative explanations have been ruled out as responsible for the emission characteristics
e.g. amplified spontaneous, directional scattering; modification of fluorescence spectrum by the cavity

- [x] Yes
- [ ] No

They have been ruled out.
The dependence of output intensity on the excitation power (see Fig. 5f, Supplementary Fig. 14(a-c(bottom ones)) and Fig. 15(b,c) exhibited an “S” shape with three distinct regions, representing the transition from spontaneous emission through amplified spontaneous emission to lasing oscillation. Hence, this clearly verifies the onset of lasing emission.

7. Theoretical analysis

Theoretical analysis that ensures that the experimental values measured are realistic and reasonable
e.g. laser threshold, linewidth, cavity gain-loss, efficiency

- [ ] Yes
- [x] No

The lasing characterization of the UCNPs-doped microring is conducted in the short-listed microring array containing four neighboring toroidal microcavity. The lasing behavior are confirmed in all four sets of microresonators. Beside, the experimental values on lasing emission are on the comparable levels with previously reported articles.
In addtion, the sensing measurement is implemented in six sets of microresonators from the same chip. The lasing threshold shift phenomenon is verified in all six samples.

8. Statistics

Number of devices fabricated and tested

- [x] Yes
- [ ] No

see Supplementary Fig. 16f.

Statistical analysis of the device performance and lifetime (time to failure)

- [ ] Yes
- [x] No

The lasing stability of UCNPs-doped WGMs lasing under nanosecond pulsed laser pumping has been investigated in previous published papers.
